# Blood eosinophil count, a marker of inhaled corticosteroid effectiveness in preventing COPD exacerbations in post-hoc RCT and observational studies: systematic review and meta-analysis

**DOI:** 10.1186/s12931-019-1268-7

**Published:** 2020-01-03

**Authors:** Timothy H. Harries, Victoria Rowland, Christopher J. Corrigan, Iain J. Marshall, Lucy McDonnell, Vibhore Prasad, Peter Schofield, David Armstrong, Patrick White

**Affiliations:** 1 Department of Public Health and Primary Care, School of Population Health & Environmental Sciences, King’s College London, 3rd floor Addison House, Guys Campus, London, SE1 1UL UK; 2 Department of Asthma Allergy & Respiratory Science, King’s College London, London, UK

**Keywords:** Pulmonary disease, chronic obstructive, Eosinophils, Inhaled corticosteroids, Randomised controlled trials, Observational studies

## Abstract

**Background:**

Blood eosinophil count has been proposed as a predictor of response to inhaled corticosteroid (ICS) in the prevention of acute exacerbations of COPD. An optimal threshold of blood eosinophil count for prescribing ICS has not been agreed. Doubt has been cast on the role by observational studies. The role of inhaled corticosteroids in this relationship, independent of long-acting bronchodilators, has not been examined.

**Methods:**

We conducted a systematic review of post-hoc analyses of randomised controlled trials (RCTs) and observational studies examining three blood eosinophil thresholds and the independent role of ICS. Included studies were categorised by the form (relative or absolute count) and cut point of eosinophil threshold used. Thresholds assessed were relative eosinophil count of 2%, and absolute counts of 150 cells/μL and 300 cells/μL. Three meta-analyses of the effect of ICS use in post-hoc analyses of RCTs based on these counts were carried out. Initial analysis included all studies of ICS vs. any non-ICS regimen. Further analysis examined the effect of ICS, independent of the effect of long-acting bronchodilators.

**Results:**

Sixteen studies examined the association between blood eosinophil count and response of exacerbation risk to ICS, in COPD patients. Eleven studies (25,881 patients) were post-hoc analyses of RCTs. Five studies (109,704 patients) were retrospective observational studies. The independent effect of ICS on the reduction of exacerbation risk was 20% at ≥2% blood eosinophil threshold (RR, 0.80; 95% CI, 0.74–0.85), 35% at ≥150 cells/μL blood eosinophil threshold (RR, 0.65; 0.52–0.79), and 39% at ≥300 cells/μL blood eosinophil threshold (RR, 0.61; 0.44–0.78). No association was found in four out of five observational studies.

**Conclusion:**

This is the first systematic review to assess, in post-hoc analyses of RCTs, the independent effect of ICS in reducing the risk of COPD exacerbation across a range of blood eosinophil thresholds. Association between ICS prescription and reduced exacerbation risk at these thresholds was confirmed. The lack of association found in the observational studies questions the relevance of these observations to a “real world” COPD population. To clarify the clinical utility of this biomarker, the association should be tested in prospective effectiveness studies.

## Introduction

Inhaled corticosteroid (ICS) therapy has been reported to be associated with a reduction in the risk of moderate and severe exacerbations in a subgroup of patients with chronic obstructive pulmonary disease (COPD) [1]. Those COPD patients with predominantly eosinophilic airways inflammation [2] may derive the most benefit from ICS use [3–7]. International guidelines reflect this targeted approach to ICS prescription [8]. The peripheral blood eosinophil count, absolute and relative, has high correlation with sputum eosinophils [9] and has gained increased recognition as a proxy of eosinophilic airways inflammation [10]. In one stable state COPD population 37.4% of patients had a blood eosinophil count of ≥2% [11]. This threshold may predict response to ICS treatment with respect to modification of exacerbation risk in COPD patients [12]. Evidence for this association has been derived from post-hoc analyses of randomised controlled trials (RCTs) [10]. High rates of prescription of ICS, outside guidelines, highlight the importance of targeted prescribing for patients with COPD. In primary care in England, 24% of COPD patients were prescribed ICS and long-acting beta-agonists (LABA) outside of the 2011 Global Initiative for Chronic Obstructive Lung Disease (GOLD) guidelines [13]. Between 2007 and 2010, large increases in ICS prescribing were not associated with expected impact on the incidence of admissions for exacerbations [14, 15].

The stability of the blood eosinophil count has been questioned, raising doubts about the appropriateness of using a single measurement as a reliable predictor of ICS response [16]. A cohort study of COPD patients with moderate airflow limitation did not find a relationship between numbers of tissue eosinophils in the airways and lung parenchyma and blood eosinophils [17]. Studies examining the role of blood eosinophils as a biomarker of ICS sensitivity have selected different thresholds of relative and absolute counts in their analyses. Previous reviews, in their comparisons of the impact on exacerbation risk of ICS vs. non-ICS, have not isolated the effect of the ICS from that of bronchodilators [7, 12]. A recent systematic review examined the impact of triple therapy vs. dual bronchodilator therapy among patients with blood eosinophilia but did not stratify the studies by eosinophil threshold [6]. An assessment of blood eosinophil count as a marker of the independent effect of ICS on reduction of exacerbation frequency would help to clarify the value of this biomarker as a guide to the prescription of these drugs.

We have chosen three thresholds as likely markers of corticosteroid-responsive disease. An association has been found between blood eosinophil counts of ≥2% and an increased risk of severe exacerbations and mortality from exacerbations among patients with COPD [18]. The benefits of oral corticosteroids have been found only in those patients with blood eosinophils of ≥2% [19]. A correlation has been reported between a relative blood eosinophil count of 2% and an absolute blood eosinophil count of 150 cells/μL [20]. The GOLD guidelines recommend the use of an absolute blood eosinophil count of ≥300 cells/μL to identify patients with COPD who should escalate their treatment from use of long-acting beta-agonist + long-acting muscarinic antagonist (LABA+LAMA) to use of LABA+ICS [8]. This systematic review provides an up-to-date analysis of the predictive value of three blood eosinophil thresholds (2%, 150 cells/μL, and 300 cells/μL) as biomarkers of the independent impact of ICS use on the risk of moderate and severe exacerbations in COPD patients. These findings are compared to those of real-world observational studies.

## Methods

The population of this review were patients with COPD stratified by blood eosinophil count. Blood eosinophil thresholds of 2%, 150 cells/μL, and 300 cells/μL were examined. The intervention was the use of inhaled corticosteroids at any dosage. The comparison was the use of ICS with any non-ICS combination inhaler or placebo (all association studies). A separate analysis examined the independent effect of ICS by comparing use of ICS monotherapy vs. placebo, or ICS + LABA vs. LABA, or ICS + LABA+LAMA vs. LABA+LAMA (ICS-independent association studies). The outcome was the risk of moderate or severe exacerbations of COPD. The report follows the Preferred Reporting Items for Systematic Reviews and Meta-analyses Statement (PRISMA) guidelines [21].

### Search strategy

Searches were made of five electronic databases from inception to 10/7/19: Medline, Cochrane Central Register of Controlled Trials, CINAHL, Embase, Web of Science. Literature search strategies were developed using medical subject headings (MeSH) and free text corresponding to obstructive airways disease, COPD, inhaled corticosteroids and eosinophils.

The web-based software Covidence was used to facilitate the screening and selection of studies. The first author (THH) conducted the database searches, removed duplicates and screened titles and abstracts of retrieved papers with respect to the eligibility criteria. Two reviewers (THH, VR) independently assessed the full text articles for eligibility. Any disagreements were resolved by discussion. If consensus was not reached, a third reviewer (PW) moderated the decision. Reasons for exclusion of full text articles were clearly recorded. Two reviewers (THH & PW) independently conducted a quality appraisal of the included studies and created the final quality appraisal by consensus.

### Inclusion criteria

**Fig. 1:**
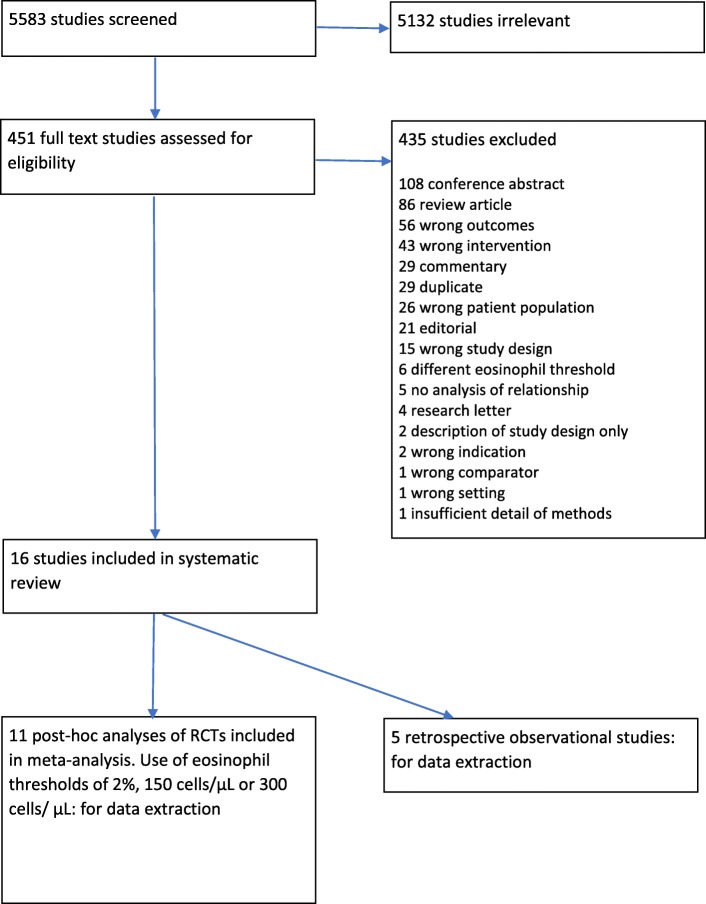
PRISMA (Preferred Reporting Items for Systematic Reviews and Meta-Analysis) flow chart

Data derived from published post-hoc analyses of randomised controlled trials (RCTs) and observational studies in primary or secondary care that reported the association, presented as a hazard ratio with confidence intervals, between blood eosinophil count, and the frequency of moderate or severe exacerbations in patients with COPD were included. Exacerbations were defined as COPD symptoms for which antibiotics or oral steroids were prescribed (moderate) or COPD symptoms for which hospital admission was recommended (severe). Included inhaled corticosteroids were beclometasone, budesonide, fluticasone (propionate and furoate), mometasone, ciclesonide, triamcinolone and flunisolide. Three blood eosinophil thresholds, 2%, 150 cells/μL and 300 cells/μL, were examined. Studies which exclusively examined other blood eosinophil thresholds were excluded, as were conference abstracts, reviews, reports and correspondence articles (Fig. 1).

### Data extraction

Data were extracted from the relevant articles and included title and authors, year of publication, treatment regimens in each study arm, annual rate of moderate and severe exacerbations and blood eosinophil threshold used.

### Quality assessment

The Cochrane Risk of Bias Tool [22] was used to assess the RCTs. The Newcastle-Ottawa scale [23] was used to assess the non-randomized observational studies. Two reviewers independently applied the risk of bias assessments to each included study. Any disagreement was resolved through discussion. We used the GRADE approach (grading of recommendations assessment, development, and evaluation) to assess the quality of the evidence for each outcome across the included studies [24].

### Meta-analysis

The meta-analysis analysed the association between ICS prescription and risk of exacerbation for each of three blood eosinophil thresholds: 2%, 150 cells/μL, 300 cells/μL. Our initial meta-analysis included studies of ICS vs. any non-ICS regimen. The comparison group may have included the effect of additional bronchodilator. Our further analysis examined the independent effect of ICS. It included studies of ICS vs. placebo or ICS + LABA vs. LABA or ICS + LABA+LAMA vs. LABA+LAMA.

We tested for between-study heterogeneity, where the true underlying effect varies between trials, using Cochran’s Q. This was quantified using the I^2^ statistic, giving the proportion of overall variation accounted for by between-study heterogeneity. We also assessed clinical and methodological heterogeneity by discussion between authors. Where we judged there was important heterogeneity, we used a random effects analysis. Otherwise, we used a fixed-effects model that assumed each study measured the same underlying effect. The protocol for this review was registered with PROSPERO, with registration ID 2019 CRD42019134833.

## Results

Five thousand five hundred eighty-three studies were identified and screened (Fig. 1 PRISMA flow chart). The full texts of 451 studies were assessed for eligibility. 16 studies fulfilled the inclusion criteria. Eleven of these studies [20, 25–34], comprising 25,881 patients, were post-hoc analyses of data from 13 RCTs (Table 1). The remaining 5 studies [35–39], comprising 109,704 patients, were retrospective observational studies (Table 2). Relevant data published from two post-hoc analyses of four RCTs were not presented in a form that could be incorporated in this review [5, 40]. The data were sought from the companies holding the data but had not been received in time for inclusion in this review. Search strategies are included see Additional file 1: Tables S3, S4, S5, S6, S7 and S8.

**Table 1:** Characteristics of included post-hoc analyses of RCTs for meta-analysis

Author	Study design	Study size (eosinophil data available)	Study arms	Eosinophil count subgroups	ICS effect isolated
Pascoe et al. [20] 2015	Two replicate double-blind, parallel group RCTs. Patients were treated with 25 μg vilanterol alone, or 25 μg vilanterol combined with 50 μg, 100 μg or 200 μg fluticasone	3177 patients (799 in 25 μg vilanterol alone group, 2378 in fluticasone + vilanterol groups)	Fluticasone + vilanterol (all doses) vs. vilanterol	< 2% and ≥ 2%; < 150 cells/μL and ≥ 150 cells/μL	Yes
Barnes et al. [25] (ISOLDE) 2016	Double-blind, parallel group, placebo-controlled RCT. Patients were treated with 500 μg fluticasone twice daily or placebo.	742 patients (370 fluticasone group, 372 placebo group).	Fluticasone vs. placebo	< 2% and ≥ 2%	Yes
Pavord et al. [26] (INSPIRE) 2016	Double-blind, double-dummy, parallel-group RCT. Patients were treated with 500 μg fluticasone + 50 μg salmeterol or 18 μg tiotropium	1269 patients (634 fluticasone + salmeterol group, 635 tiotropium group)	Fluticasone + salmeterol vs. Tiotropium	< 2% and ≥ 2%	No
Pavord et al. [26] (TRISTAN I) 2016	Double-blind, parallel-group RCT. Patients were treated with 500 μg fluticasone + 50 μg salmeterol or 50 μg salmeterol	696 patients (341 fluticasone + salmeterol group, 355 salmeterol group)	Fluticasone + salmeterol vs. salmeterol	< 2% and ≥ 2%	Yes
Pavord et al. [26] (TRISTAN II) 2016	Double-blind, parallel-group, placebo-controlled RCT. Patients were treated with 500 μg fluticasone or placebo	707 patients (360 fluticasone group, 347 placebo group)	Fluticasone vs. placebo	< 2% and ≥ 2%	Yes
Pavord et al. [26] (SCO30002) 2016	Double-blind, parallel-group, placebo-controlled RCT. Patients were treated with 500 μg fluticasone or placebo	244 patients (124 fluticasone group, 120 placebo group)	Fluticasone vs. placebo	< 2% and ≥ 2%	Yes
Watz et al. [27] (WISDOM) 2016	Double-blind, parallel-group RCT. Patients treated with 18 μg tiotropium daily plus 500 μg fluticasone + 50 μg salmeterol twice daily for 6 weeks. Then randomised to withdrawal of fluticasone or continued triple therapy.	2296 patients (1144 ICS-continuation group, 1152 ICS-withdrawal group)	Fluticasone + tiotropium + salmeterol vs. tiotropium + salmeterol	< 2% and ≥ 2%; < 150 cells/μL and ≥ 150 cells/μL; < 300 cells/μL and ≥ 300 cells/μL	Yes
Papi et al. [28] (EFFECT) 2017	Double-blind, parallel-group RCT. Patients were treated with 500 μg fluticasone + 20 μg formoterol twice daily or formoterol 12 μg twice daily.	1177 patients (587 fluticasone + formoterol group, 590 formoterol group)	Fluticasone + formoterol vs. formoterol	< 2% and ≥ 2%	Yes
Vestbo et al. [29] (TRINITY) 2017	Double-blind, parallel-group RCT. Patients were treated with 100 μg beclometasone + 6 μg formoterol + 12.5 μg glycopyrronium two puffs twice daily or 18 μg tiotropium	2153 patients (1077 fixed triple group, 1076 tiotropium group)	Beclometasone + formoterol + glycopyrronium vs. tiotropium	< 2% and ≥ 2%	No
Roche et al. [30] (FLAME) 2017	Double-blind, parallel-group RCT. Patients were treated with 500 μg fluticasone + 50 μg salmeterol twice daily or 110 μg indacaterol + 50 μg glycopyrronium once a day	3349 patients (1677 fluticasone + salmeterol group, 1672 indacaterol + glycopyrronium group)	Fluticasone + salmeterol vs. indacaterol + glycopyrronium	< 2% and ≥ 2%; < 300 cells/μL and ≥ 300 cells/μL	No
Chapman et al. [31] (SUNSET) 2018	Double-blind, triple-dummy, parallel-group RCT. Patients were treated with 18 μg tiotropium once daily plus 500 μg fluticasone + 50 μg salmeterol twice daily or 110 μg indacaterol + 50 μg glycopyrronium once a day	1051 patients (526 fluticasone + salmeterol + tiotropium group, 527 indacaterol + glycopyrronium group)	Fluticasone + tiotropium + salmeterol vs. indacaterol + glycopyrronium	< 2% and ≥ 2%; < 300 cells/μL and ≥ 300 cells/μL	Yes
Papi et al. [32] (TRIBUTE) 2018	Double-blind, double-dummy, parallel-group RCT. Patients were treated with 87 μg beclometasone + 5 μg formoterol + 9 μg glycopyrronium two puffs twice daily or 85 μg indacaterol + 43 μg glucopyrronium once a day	1532 patients (764 BDP/FF/G, 768 IND/GLY)	Beclometasone/formoterol/glycopyrronium vs. indacaterol/ glycopyrronium	< 2% and ≥ 2%	Yes
Ferguson et al. [33] (KRONOS) 2018	Double-blind, parallel-group RCT. Patients were treated with 320 μg budesonide + 18 μg glycopyrolate + 9.6 μg formoterol two puffs twice daily or 18 μg glycopyrolate + 9.6 μg formoterol two puffs twice daily	1267 patients (640 BGF, 627 GFF)	Budesonide/glycopyrolate/formoterol vs. glycopyrolate/formoterol	< 150 cells/μL and ≥ 150 cells/μL	Yes
Lipson et al. [34] (IMPACT) 2018	Double-blind, parallel-group RCT. Patients were treated with 100 μg fluticasone + 62.5 μg umeclidinium + 25 μg vilanterol once daily or 62.5 μg umeclidinium + 25 μg vilanterol once daily	6221 patients (4151 triple therapy, 2070 umeclidinium + vilanterol)	Fluticasone furoate/umeclidinium/ vilanterol vs. umeclidinium/vilanterol	< 150 cells/μL and ≥ 150 cells/μL	Yes

**Table 2:** Characteristics of included observational studies

Study	Inclusion criteria	Exclusion criteria	Disease severity	Exacerbation history	LAMA use at/prior to enrolment	ICS use at/prior to enrolment	Eosinophils measured
Song 2017 [35] 168 patients	> 40 years. Smoked > 10 years. FEV1/FVC < 0.7	Asthma. FEV1 reversibility > 12%. Hx atopy, allergic rhinitis. Blood eos > 5% IgE > 100	FEV1% predicted 55.5 ± 18	Mean 31.9% patients AECOPD in 12 months prior to study enrolment. Recorded 6-monthly patient reviews	59%	43%	Not stated
Suissa 2018 [36] 24,732 patients	≥55 years. COPD new users of LAMA or LABA+ICS from 1.1.02. Blood eosinophil count before cohort entry	Initiated on LAMA & LABA+ICS at same time. Asthma NOT excluded	Not known	Record drawn from READ code. Mean 33.6% patients had ≥1 AECOPD in 12 months prior to study enrolment	0%	0%	Prior to study entry.
Oshagbemi 2018 [37] 32,693 patients	≥40 year. New diagnosis COPD from 1.1.05	Asthma. Any previous AECOPD. ICS use in past year	Not known	Record drawn from READ code	14%	0%	Single eosinophil measure at point closest to index date (start of follow-up)
Oshagbemi 2019 [38] 48,157 patients	≥40 year. New diagnosis COPD from 1.1.05	Asthma. AECOPD in 6 months prior to index date	Not known	Record drawn from READ code	18%	28%	Single eosinophil measure at point closest to index date (start of follow-up)
Suissa 2019 [39] 3954 patients	≥55 years. new use LAMA or LABA+ICS from 1.1.02. Blood eosinophil count before cohort entry	Asthma	FEV1% predicted 53.9% (LABA + LAMA) 52.7% (LABA + ICS)	Record drawn from READ code. Mean 41% of patients had ≥1 AECOPD in 12 months prior to study enrolment	77% (LABA+LAMA) 49.1% (LABA + ICS)	37% (LABA+LAMA) 52.8% (LABA+ICS)	Baseline

The post-hoc analyses of RCTs were divided into those which had balanced treatment arms except for ICS, and those where one arm received an additional treatment (typically a LAMA). The independent effect of the ICS on risk of moderate or severe exacerbation was isolated in the RCTs which had balanced treatment arms [20, 25–28, 31–34] but not in those which did not [26, 29, 30]. One study [26] reported the results of three different RCTs: INSPIRE, TRISTAN and SCO30002. The INSPIRE study did not isolate the independent effect of the ICS whereas the TRISTAN and SCO30002 studies did. The TRISTAN study comprised five different drug comparisons. Two of these, Fluticasone propionate (FP) + Salmeterol (SAL) vs. SAL and FP vs. placebo, isolated the independent effect of the ICS. The patient groups in these two drug comparisons were mutually exclusive. These have been labelled here as TRISTAN I (FP/SAL vs. SAL) and TRISTAN II (FP vs. placebo) and treated as two separate investigations in the meta-analysis.

### Association between impact of ICS and blood eosinophil threshold

**Fig. 2:**
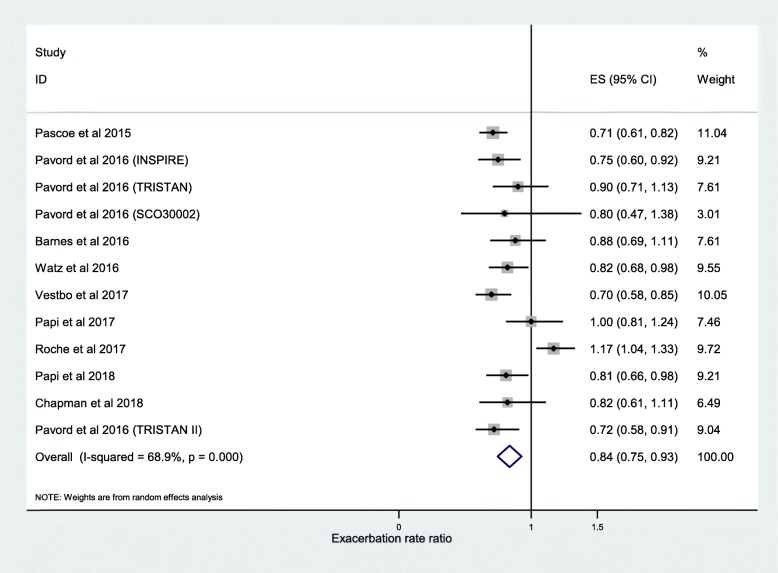
Risk ratio exacerbations COPD patients receiving ICS vs. non-ICS treatment ≥2% eosinophils (all association studies). ES, effect size

**Fig. 3:**
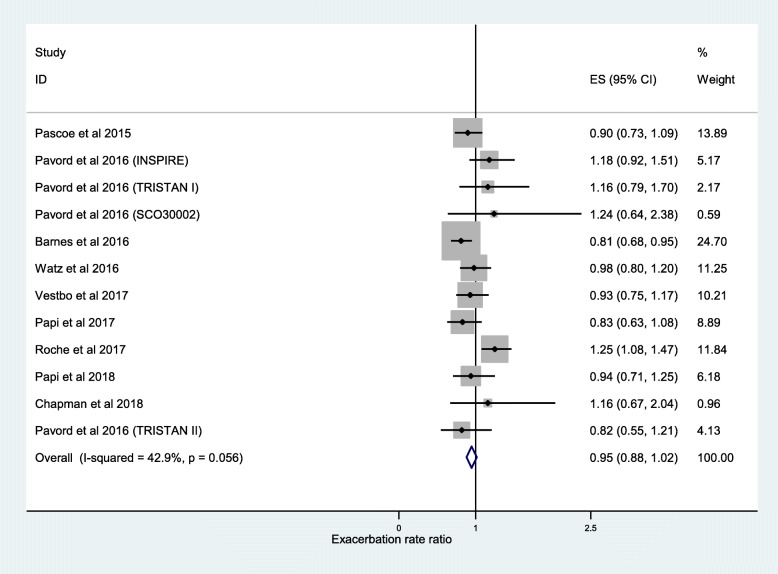
Risk ratio exacerbations COPD patients receiving ICS vs. non-ICS treatment < 2% eosinophils (all association studies). ES, effect size

**Fig. 4:**
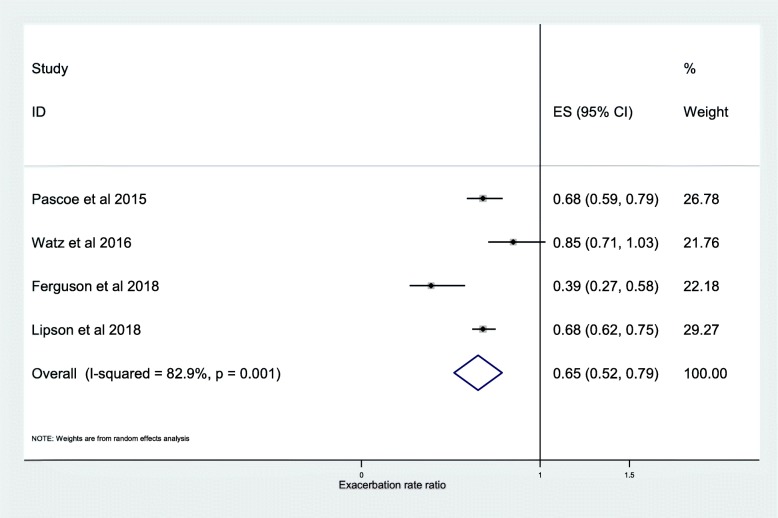
Risk ratio exacerbations COPD patients receiving ICS vs. non-ICS treatment ≥150cells/μL eosinophils (ICS-independent association studies). ES, effect size

**Fig. 5:**
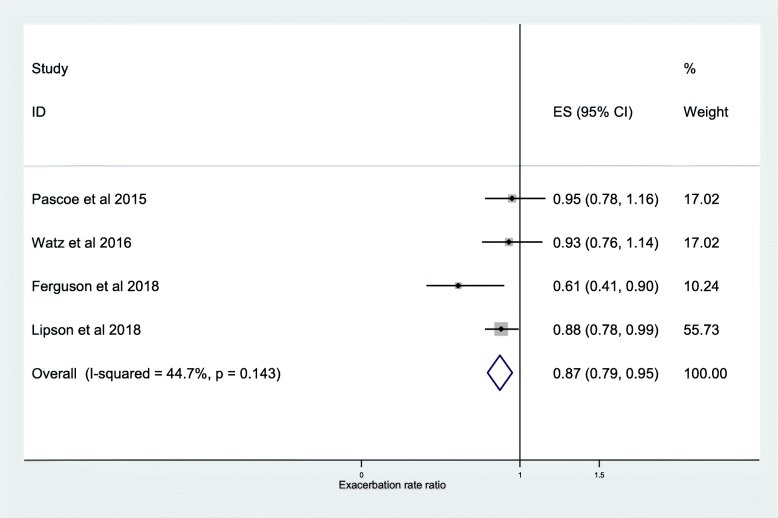
Risk ratio exacerbations COPD patients receiving ICS vs. non-ICS treatment < 150cells/μL eosinophils (ICS-independent association studies). ES, effect size

The results of 11 studies taken from the post-hoc analyses of 13 RCTs were pooled for meta-analysis of the association between frequency of moderate or severe exacerbations and ICS use. We found that ICS treatment reduced exacerbations compared with no ICS for patients with ≥2% blood eosinophils (RR, 0.84; 95% CI, 0.75–0.93) (Fig. 2). For patients with < 2% blood eosinophils we found no significant difference (RR, 0.95; 95% CI, 0.88–1.02) (Fig. 3). A significant difference was also observed at ≥150 cells/μL blood eosinophil threshold (RR, 0.65; 95% CI, 0.52–0.79) (Fig. 4) and at < 150 cells/μL blood eosinophil threshold (RR, 0.87; 95% CI, 0.79–0.95) (Fig. 5) but not at ≥300 cells/μL blood eosinophil threshold (RR, 0.76; 95% CI, 0.43–1.09) (Additional file 1: Figure S1).

**Fig. 6:**
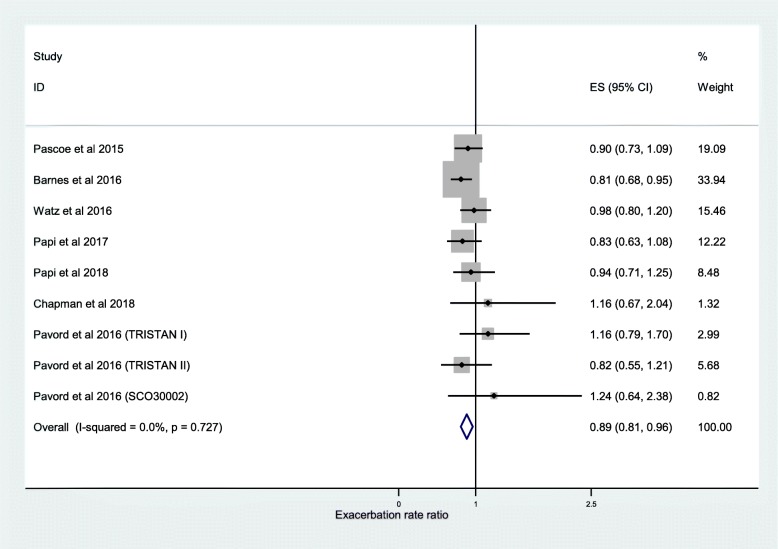
Risk ratio exacerbations COPD patients receiving ICS vs. non-ICS treatment < 2% eosinophils (ICS-independent association studies). ES, effect size

**Fig. 7:**
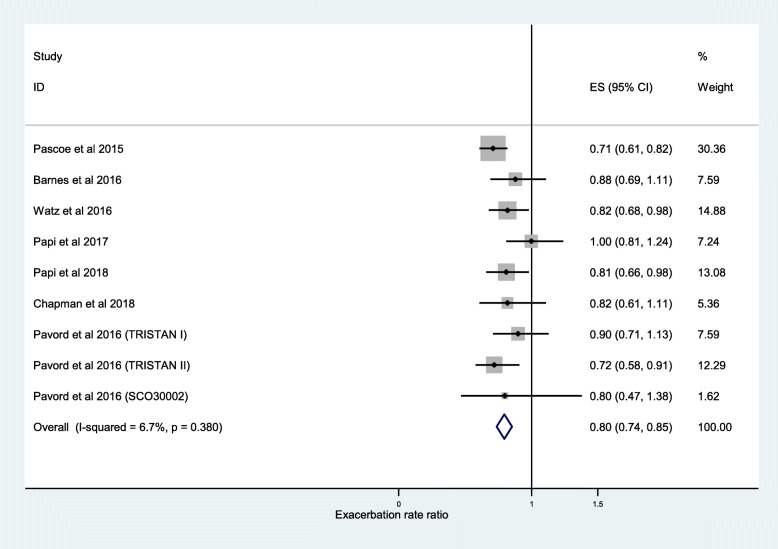
Risk ratio exacerbations COPD patients receiving ICS vs. non-ICS treatment ≥2% eosinophils (ICS-independent association studies). ES, effect size

**Fig. 8:**
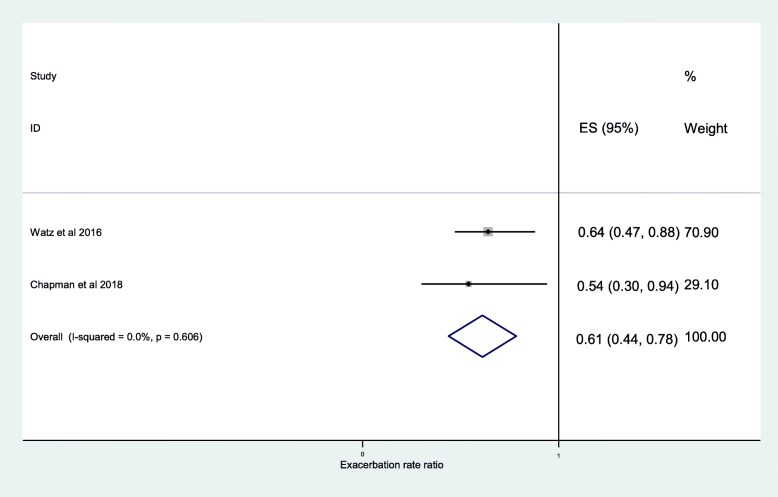
Risk ratio exacerbations COPD patients receiving ICS vs. non-ICS treatment ≥300cells/μL eosinophils (ICS-independent association studies). ES, effect size

**Fig. 9:**
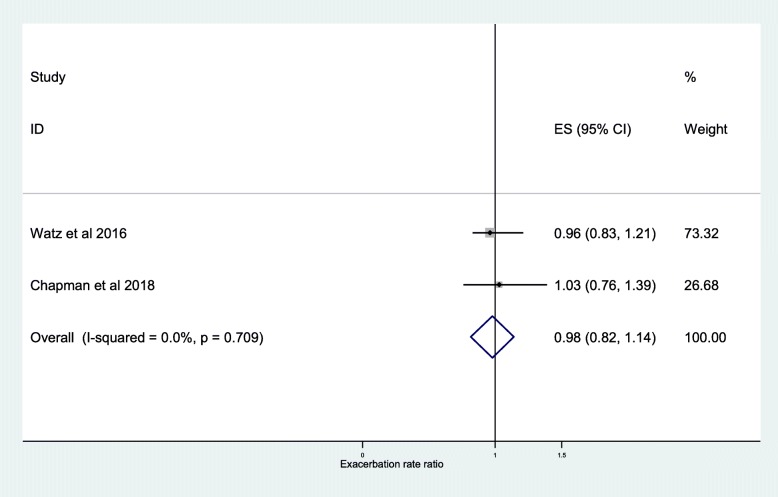
Risk ratio exacerbations COPD patients receiving ICS vs. non-ICS treatment <300cells/μL eosinophils (ICS-independent association studies). ES, effect size

The further analysis, among the studies that described the independent association between exacerbation frequency and ICS, demonstrated a significant difference between ICS treatment and non-ICS treatment at < 2% blood eosinophil threshold (RR, 0.89; 95% CI, 0.81–0.97) (Fig. 6) and < 150 cells/μL blood eosinophil threshold (RR, 0.87; 95% CI, 0.79–0.95) (Fig. 5). The independent effect of ICS on reduction in frequency of moderate or severe exacerbations was 20% at ≥2% blood eosinophil threshold (RR, 0.80; 95% CI, 0.74–0.85) (Fig. 7), 35% at ≥150 cells/μL blood eosinophil threshold (RR, 0.65; 95% CI, 0.52–0.79) (Fig. 4), and 39% at ≥300 cells/μL blood eosinophil threshold (RR, 0.61; 95% CI, 0.44–0.78) (Fig. 8). No difference was seen at < 300 cells/μL blood eosinophil threshold (RR, 0.98; 95% CI, 0.82–1.14) (Fig. 9).

At the 2% blood eosinophil threshold we have presented the results of the pooled analysis of all studies and the further analysis of those studies which show the independent effect of ICS. We have presented only the results of the further analysis at the blood eosinophil thresholds of 150 cells/μL and 300 cells/μL. The results of the meta-analysis of all studies at ≥300 cells/μL blood eosinophil threshold, including those that did not isolate the independent effect of ICS, are given in the Additional file 1: Figure S1. The positive association and strength of the effect size was greatest when the independent effect of ICS was examined at the blood eosinophil thresholds of 150 cells/μL and 300 cells/μL. We used a random-effects model, to calculate the pooled estimate accounting for heterogeneity, where we judged there was important heterogeneity. In each of these cases *p* < 0.1 or I^2^ > 50%. Funnel plots from each analysis are presented in Additional file 1: Figures S2, S3, S4, S5, S6, S7, S8, S9 and S10.

### Observational studies

Suissa et al. reported that patients with blood eosinophils of ≥4% who were initiated on LABA+ICS had 21% fewer moderate or severe exacerbations noted in the record compared to those patients initiated on a LAMA (RR, 0.79; 95% CI, 0.70–0.88) [36]. In the same study, in patients with blood eosinophils of ≥300/μL the risk of an exacerbation noted in the record was 24% (RR, 0.76; 95% CI, 0.67–0.85) lower. No association was found between blood eosinophil count and impact of ICS on exacerbation frequency in any of the other observational studies [35, 37–39].

### Quality assessment

The risk of bias assessment of the post-hoc analysis of the RCTs is presented in Additional file 1: Table S1*.* The RCTs were at low risk of bias for random sequence generation and performance bias. Five studies had unclear detection bias and six studies had either unclear or high risk of attrition bias. Six studies had unclear risk of reporting bias. The GRADE assessment identified the quality of evidence for the association between blood eosinophil count and the response of exacerbation risk to ICS, in COPD patients as low at blood eosinophil thresholds ≥2% and ≥ 150 cells/μL and as very low at blood eosinophil threshold ≥300 cells/μL (Additional file 1: Table S2). The results of the quality assessment of the observational studies are presented in Additional file 1: Table S3. The studies were of a uniform high quality.

## Discussion

Our meta-analysis has identified a positive association between use of ICS and risk of moderate or severe COPD exacerbations when stratified by blood eosinophil threshold. When all studies were examined the positive association was present at ≥2% and ≥ 150 cells/μL eosinophil thresholds, but not at the ≥300 cells/μL threshold. When the independent effect of ICS was isolated the association was positive at each of the three thresholds and the degree of the association/effect size had increased. The evidence for the association at each of these thresholds was low or very low.

A lack of association between use of ICS and risk of moderate or severe COPD exacerbations was found in 4 of the 5 observational studies [35, 37–39]. Reasons for the difference in results between the two types of studies may include differences in patient demographics, or differences between bronchodilator use by participants of the RCTs compared to those of the observational studies. The results of the observational studies may be less reliable than those of the RCTs due to differences in quality between the two types of studies. It has been estimated that those patients with COPD selected for clinical trials are representative of about 7% of the entire COPD population [41].

Eleven of the 13 RCTs recruited only patients with a history of ≥1 exacerbation in the past 12 months. In three of the observational studies [35, 36, 39] the proportion of patients reported to have experienced ≥1 exacerbation in the past 12 months was between 32 & 41%. The remaining two observational studies excluded patients who had experienced an exacerbation either in the 6 months prior to the index date [38] or at any point in the past [37]. COPD patients with the greatest degree of airflow impairment may experience the largest number of exacerbations. All of the RCTs recruited patients with either severe to very severe airflow limitation [27, 32] or moderate to severe airflow limitation. In 3 of the 5 observational studies the disease severity of the patients was unknown [36–38]. Participants in the remaining 2 observational studies had a mean FEV1% predicted of > 50% [35, 39]. The severity of airflow limitation of patients in the RCTs is likely to have been greater and their baseline exacerbation prevalence higher than those in the observational studies. The effect of ICS use is likely to be more apparent within the RCTs in comparison to the observational studies.

One potential explanation of the disparity in results could be a higher rate of undiagnosed asthma among the participants of the RCTs compared with the observational studies. Patients with a current diagnosis of asthma were excluded in all but one of the RCTs [20, 26–34]. Three RCTs recruited patients who may have had a past diagnosis of asthma [20, 28, 34]. Eleven RCTs undertook reversibility testing [20, 25, 26, 29–34] and 4 of these RCTs excluded patients with a reversibility of > 10% [25, 26]. The remaining 7 RCTs [20, 29–34] included patients with reversibility, an indicator of asthma, their prevalence ranging from 10 to 25%. In 4 of 5 observational studies participants with an asthma diagnosis were excluded [35, 37–39]. The one observational study that did not exclude patients with asthma found a positive association at blood eosinophil thresholds ≥4% and ≥ 300 cells/μL between use of LABA+ICS and a decrease in exacerbation frequency, compared to use of LAMA [36]. It is probable that both the RCTs and observational studies included a proportion of patients with asthma/reversibility, in whom the association between ICS use and reduction in exacerbation risk would be strongest. A baseline blood eosinophil count of ≥500 cells/μL has been identified in 30% of patients with asthma [42] but in only 3.3% of patients with COPD [17]. The FLAME study, one of the few RCTs which failed to find an association between eosinophil count and impact of ICS in COPD patients, excluded those patients with blood eosinophils ≥600 cells/μL [30].

Use of concurrent bronchodilators among patients in the observational studies, but not those of the RCTs, may have contributed to the lack of association found between ICS use and risk of exacerbations in the observational studies. The findings of the observational studies raise questions about the transferability of the post-hoc RCT findings to real world clinical practice. A strong association between impact of ICS and risk of exacerbations at the three eosinophil thresholds was evident from the meta-analysis. Any new prospective trial with randomization by eosinophil status is unlikely to come up with an assessment of efficacy of ICS in preventing exacerbations in COPD in patients above the experimental threshold that gives a different result. However, the observational studies do suggest that the effectiveness of ICS using the eosinophil threshold of 2% (or 150 cells/μL) in a real-world setting cannot be assumed from efficacy trials. An effectiveness trial, which excludes patients with asthma, may help in answering this question. A limitation of the observational studies is the lack of consistency in the reporting of concurrent bronchodilator use among participants. However, it is exactly these factors together with differences in clinical and demographic characteristics that make effectiveness trials so important in testing the applicability of efficacy trials which have such tight inclusion and exclusion criteria as drug trials in COPD.

This review has examined the predictive value of blood eosinophils on the impact of ICS when patients are stratified by both relative and absolute blood eosinophil counts. The relative blood eosinophil count will be altered by the numbers of other cells within the white blood cell population. This suggests that the relative blood eosinophil count is inherently less reliable than the absolute blood eosinophil count. Despite this we have identified a positive association at raised thresholds of both the relative and absolute blood eosinophil counts.

GOLD guidelines recommend escalation from dual LABA+LAMA therapy to LABA+ICS in patients with blood eosinophil counts ≥300 cells/μL or in those with blood eosinophils of ≥100 cells/μL and a history of ≥2 exacerbations or 1 severe exacerbation [8]. The meta-analysis by Oshagbemi et al. identified a positive association between ICS use and reduction in exacerbation risk at blood eosinophil thresholds of ≥100 to ≥340 cells/μL [7]. They did not stratify the results into narrower eosinophil ranges, nor did they differentiate by past exacerbation history. The effect, or lack of effect of ICS on exacerbation risk at low eosinophil thresholds may have been over-ridden by the effect seen at higher eosinophil thresholds. Their findings confirm the GOLD recommendation for use of ICS at eosinophil threshold ≥300 cells/μL but do not directly justify use at eosinophil threshold ≥100 cells/μL accompanied by a history of ≥2 exacerbations or 1 severe exacerbation. Bafadhel et al. described a continuous relationship between blood eosinophil count and the impact of budesonide on reduction in exacerbation risk [5]. At eosinophil counts of < 200 cells/μL the confidence intervals were broad and the upper limit close to one.

The blood eosinophil count may help characterize the nature of exacerbations within a population of patients with COPD who experience exacerbations. Its value is less clear for those patients with COPD who do not have exacerbations. The eosinophil count may be elevated for other reasons including allergies, inflammatory conditions and malignancies. In addition, a single blood eosinophil count may be an unsatisfactory basis upon which to prescribe ICS.

## Conclusion

This meta-analysis has demonstrated a positive association and a strong effect size, across three blood eosinophil thresholds – 2%, 150 cells/μL and 300 cells/μL, between ICS, independently assessed, and the risk of an exacerbation of COPD. The strength of the association was less when the contributory effects of the bronchodilators were not excluded. The strength of the association increased as the blood eosinophil threshold increased. The quality of the evidence for these associations was low or very low. Data from the post-hoc RCTs may not justify extrapolation to the general COPD population. The lack of association found in the observational studies suggests that this relationship may not be present within the “real world” COPD population. A prospective effectiveness study which differentiates patients according to past exacerbation history and reversibility of lung function and stratified by ranges of eosinophil counts rather than eosinophil thresholds should be conducted.

## Supplementary information

**Additional file 1: Figure S1.** Forest plot of risk ratio exacerbations COPD patients receiving ICS vs. non-ICS treatment ≥300cells/μL eosinophils (all association studies). **Figures S2 to S10.** Funnel plots of all studies for each of the outcomes at the three eosinophil thresholds. **Table S1.** Risk of bias summary of post-hoc RCTs. **Table S2.** GRADE assessment of outcomes from post-hoc RCTs. **Table S3.** Newcastle-Ottawa Quality Assessment Scale for Observational Cohort Studies. **Tables S4 to S8.** Database search results.

## Data Availability

All data generated or analysed during this study are included in this published article [and its supplementary information files].

## Abbreviations

COPD: Chronic obstructive pulmonary disease
FP: Fluticasone propionate
GOLD: Global Initiative for Chronic Obstructive Lung Disease
GRADE: Grading of recommendations assessment, development, and evaluation
ICS: Inhaled corticosteroids
LABA: Long-acting beta-agonist
LAMA: Long-acting muscarinic antagonist
MeSH: Medical Subject Heading
PRISMA: Preferred Reporting Items for Systematic Reviews and Meta-Analysis
RCT: Randomised controlled trial
SAL: Salmeterol
