# Supplementary material for: Blood eosinophil count, a marker of inhaled corticosteroid effectiveness in preventing COPD exacerbations in post-hoc RCT and observational studies: systematic review and meta-analysis
Source: Respir Res. 2020 Jan 3;21:3. doi: 10.1186/s12931-019-1268-7 (PMC6942335; doi:10.1186/s12931-019-1268-7)
Supplement: Supplementary file 1 — Additional file 1: Figure S1. Forest plot of risk ratio exacerbations COPD patients receiving ICS vs. non-ICS treatment ≥300cells/μL eosinophils (all association studies). Figures S2 to S10. Funnel plots of all studies for each of the outcomes at the three eosinophil thresholds. Table S1. Risk of bias summary of post-hoc RCTs. Table S2. GRADE assessment of outcomes from post-hoc RCTs. Table S3. Newcastle-Ottawa Quality Assessment Scale for Observational Cohort Studies. Tables S4 to S8. Database search results. [file 12931_2019_1268_MOESM1_ESM.docx]

**Blood eosinophil count, a marker of inhaled corticosteroid effectiveness in preventing COPD exacerbations in post-hoc RCT and observational studies: systematic review and meta-analysis**

Timothy H Harries^1*^, Victoria Rowland^1^, Christopher J Corrigan^2^, Iain J Marshall^1^,

Lucy McDonnell^1^, Vibhore Prasad^1^, Peter Schofield^1^, David Armstrong^1^,

Patrick White^1^

**Additional file**

**Figure S1**

**Risk ratio exacerbations COPD patients receiving ICS vs. non-ICS treatment ≥300cells/µL eosinophils (all association studies).** ES, effect size

**Figure S2**

**Funnel plot of studies comparing Risk ratio exacerbations COPD patients receiving ICS vs. non-ICS treatment <2% eosinophils (all association studies).**

**Figure S3**

**Funnel plot of studies comparing risk ratio exacerbations COPD patients receiving ICS vs. non-ICS treatment ≥2% eosinophils (all association studies).**

**Figure S4**

**Funnel plot of studies comparing risk ratio exacerbations COPD patients receiving ICS vs. non-ICS treatment <2% eosinophils (ICS-independent association studies).**

**Figure S5**

**Funnel plot of studies comparing risk ratio exacerbations COPD patients receiving ICS vs. non-ICS treatment ≥2% eosinophils (ICS-independent association studies).**

**Figure S6**

**Funnel plot of studies comparing risk ratio exacerbations COPD patients receiving ICS vs. non-ICS treatment <150cells/µL eosinophils (ICS-independent association studies).**

**Figure S7**

**Funnel plot of studies comparing risk ratio exacerbations COPD patients receiving ICS vs. non-ICS treatment ≥150cells/µL eosinophils (ICS-independent association studies).**

**Figure S8**


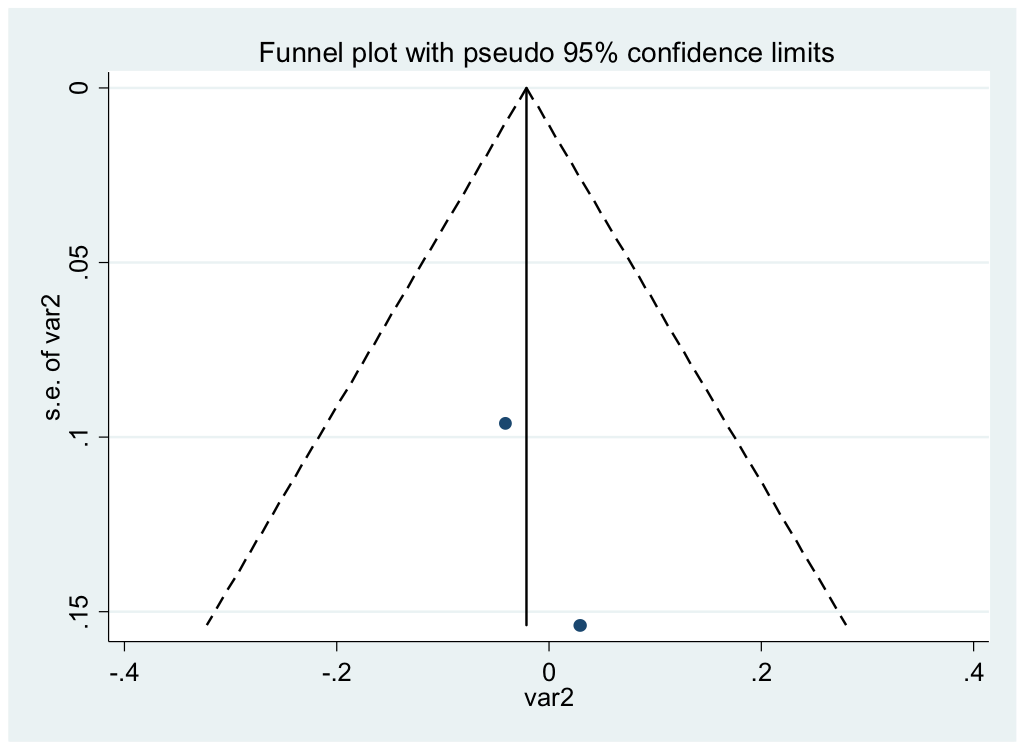


**Funnel plot of studies comparing risk ratio exacerbations COPD patients receiving ICS vs. non-ICS treatment <300cells/µL eosinophils (ICS-independent association studies).**

**Figure S9**


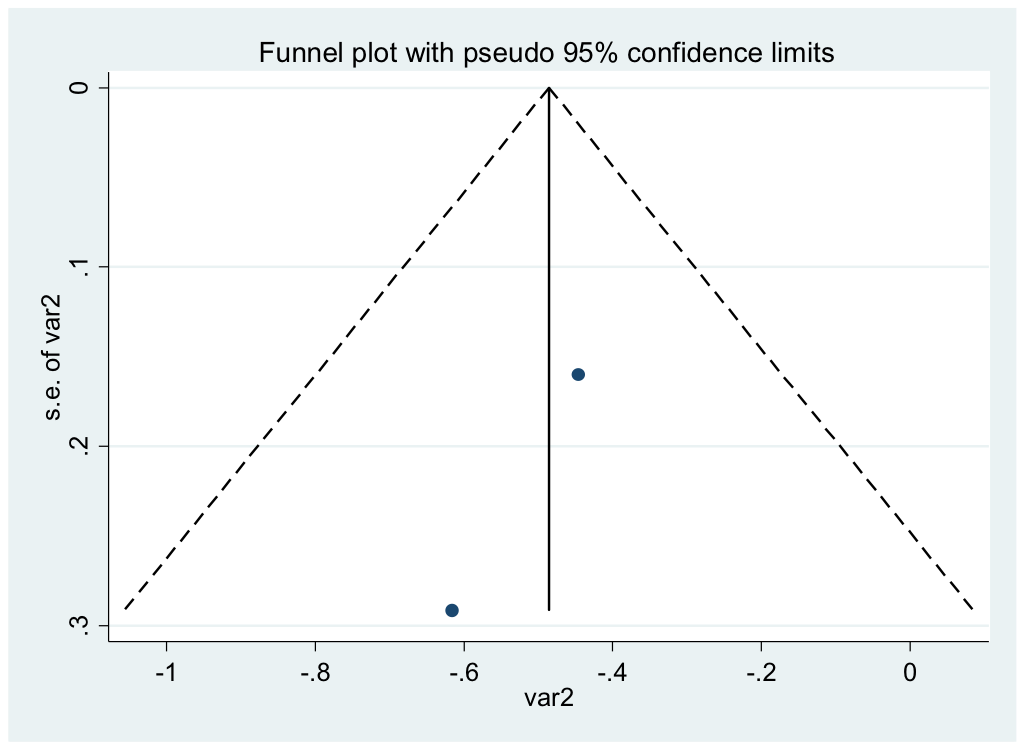


**Funnel plot of studies comparing risk ratio exacerbations COPD patients receiving ICS vs. non-ICS treatment ≥300cells/µL eosinophils (ICS-independent association studies).**

**Figure S10**

**Funnel plot of studies comparing risk ratio exacerbations COPD patients receiving ICS vs. non-ICS treatment ≥300cells/µL eosinophils (all association studies).**

**Table S1 Risk of bias summary of post-hoc RCTs.**


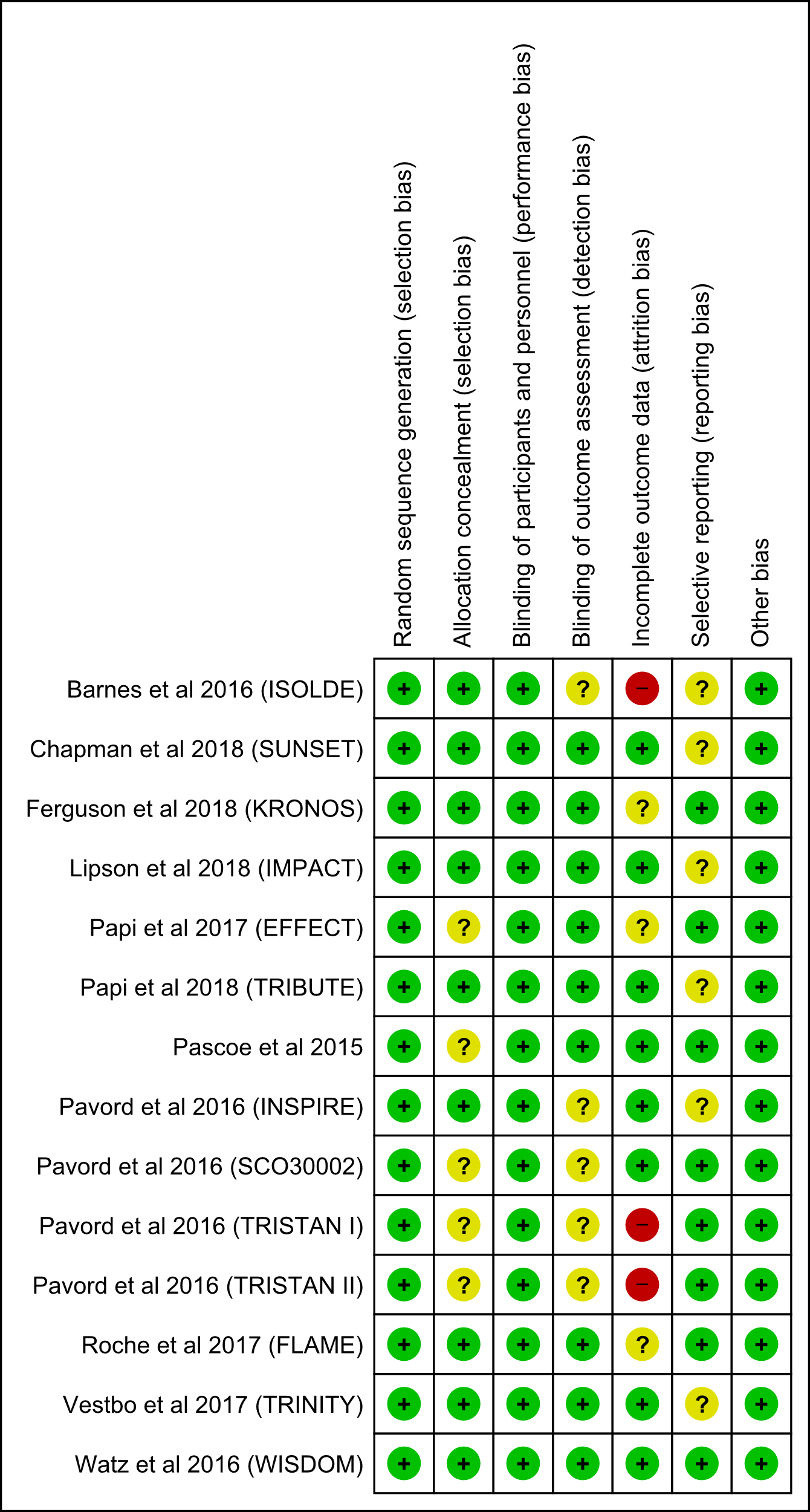


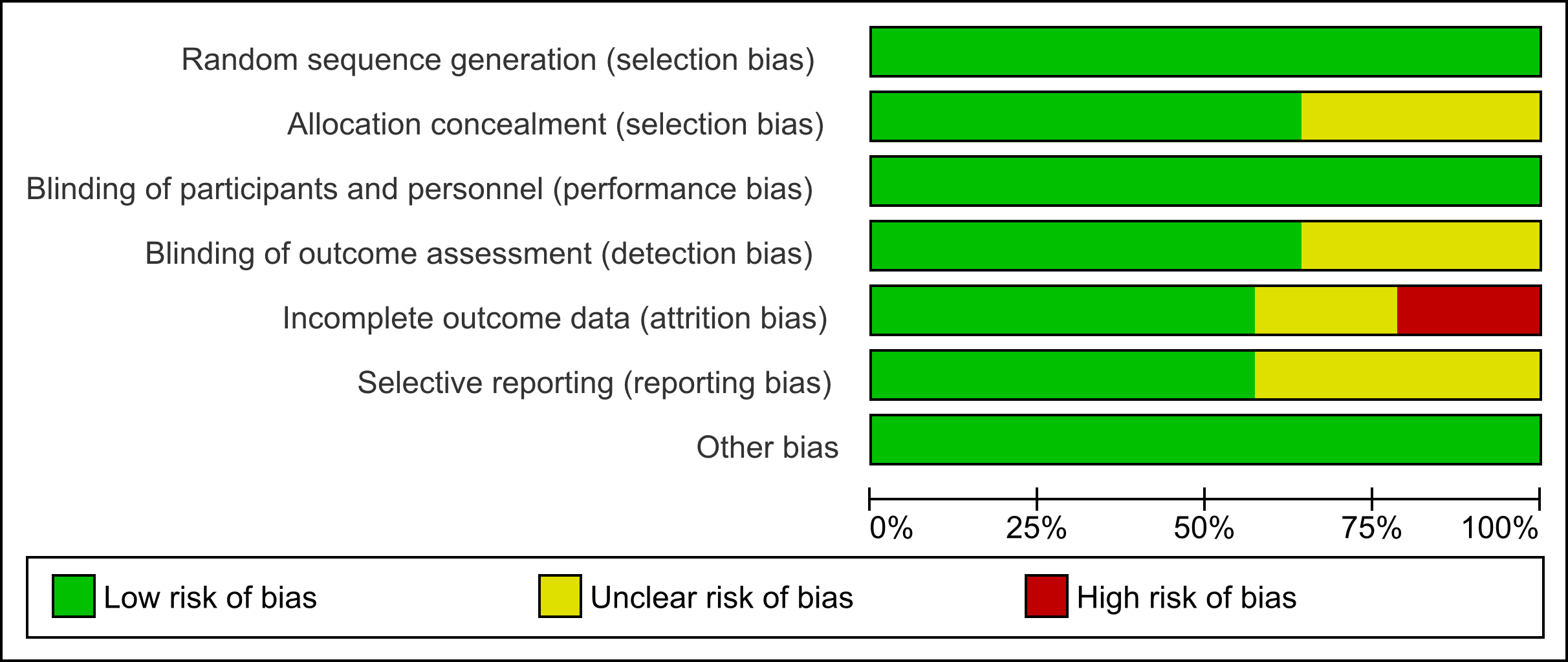


**Table S2 GRADE assessment of outcomes from post-hoc RCTs**

| Threshold | Studies (pts) | Design | Limitations (ROB) | Indirectness of patients, intervention and comparator | Inconsistency | Imprecision | Publication bias | Quality of evidence |
| --- | --- | --- | --- | --- | --- | --- | --- | --- |
| <2% | 11 (7101) | Post-hoc RCT | 0 | 0 | -1 | -1 | -1 | Very low + |
| ≥2% | 11 (11356) | Post-hoc RCT | 0 | 0 | -1 | 0 | -1 | Low ++ |
| <150/µL | 4 (5784) | Post-hoc RCT | 0 | 0 | -1 | 0 | -1 | Low ++ |
| ≥150/µL | 4 (7731) | Post-hoc RCT | 0 | 0 | -1 | 0 | -1 | Low ++ |
| <300/µL | 3 (5161) | Post-hoc RCT | 0 | 0 | 0 | -1 | -1 | Low ++ |
| ≥300/µL | 3 (1427) | Post-hoc RCT | 0 | 0 | -1 | -1 | -1 | Very low + |

**Table S3 Newcastle-Ottawa Quality Assessment Scale for Observational Cohort Studies**

| **Author** | **Selection of exposed cohort^a^** | **Selection of non-exposed cohort^b^** | **Ascertainment of exposure^c^** | **Outcome at start of study^d^** | **Comparability of cohorts^e^** | **Assessment of outcome^f^** | **Follow-up  of sufficient duration^g^** | **Adequate follow-up of cohorts^h^** | **Overall  Score** |
| --- | --- | --- | --- | --- | --- | --- | --- | --- | --- |
| **Song 2016** (31) | b) (+1) | a) (+1) | b) (+1) | a) (+1) | a) & b) (+2) | c) (+0) | a) (+1) | d) (+0) | 7 |
| **Suissa 2018** (32) | a) (+1) | a) (+1) | a) (+1) | a) (+1) | a) & b) (+2) | b) (+1) | a) (+1) | b) (+1) | 9 |
| **Oshagbemi 2018** (33) | a) (+1) | a) (+1) | a) (+1) | a) (+1) | a) & b) (+2) | b) (+1) | a) (+1) | b) (+1) | 9 |
| **Oshagbemi 2019** (34) | b) (+1) | a) (+1) | a) (+1) | a) (+1) | a) & b) (+2) | b) (+1) | a) (+1) | b) (+1) | 9 |
| **Suissa 2019**  (35) | a) (+1) | a) (+1) | a) (+1) | a) (+1) | a) & b) (+2) | b) (+1) | a) (+1) | b) (+1) | 9 |

**^a^ Selection of exposed cohort:** a) truly representative of the average population with COPD of interest in the community (1 point), b) somewhat representative of the average population with COPD in the community (1 point), c) selected group of users eg nurses, volunteers (0 points), d) no description of the derivation of the cohort (0 points).

**^b^** **Selection of non-exposed cohort:** a) drawn from the same community as the exposed cohort (1 point), b) drawn from a different source (0 points), c) no description of the derivation of the non-exposed cohort (0 points).

**^c^ Ascertainment of exposure:** a) secure record (1 point), b) structured interview (1 point), c) written self-report (0 points), d) no description (0 points).

**^d^ Demonstration That Outcome of Interest Was Not Present at Start of Study:** a) yes (1 point), b) no (0 points).

**^e^ Comparability of cohorts:** a) study controls for age (1 point), b) study controls for gender (1 point).

**^f^ Assessment of outcome:** a) independent blind assessment (1 point), b) record linkage (1 point), c) self-report (0 points), d) no description (0 points).

**^g^ Follow-up long enough for outcomes to occur:** a) yes (1 point), b) no (0 points).

**^h^ Adequacy of follow up of cohorts:** a) complete follow up - all subjects accounted for (1 point), b) subjects lost to follow up unlikely to introduce bias - small number lost - > 75% follow up, or description provided of those lost (1point), c) follow up rate < 75%% and no description of those lost (0 points), d) no statement (0 points).

**Database searches**

**Table S4 Medline search strategy (Ovid MEDLINE 1946 to July 10, 2019)**

| 1 | exp Pulmonary Disease, Chronic Obstructive/ | 48883 |
| --- | --- | --- |
| 2 | exp Lung Diseases, Obstructive/ | 196489 |
| 3 | emphysema$.mp. | 33746 |
| 4 | copd.mp. | 39389 |
| 5 | coad.mp. | 270 |
| 6 | cobd.mp. | 18 |
| 7 | (obstruct$ adj3 (pulmonary or lung$ or airway$ or airflow$ or bronch$ or respirat$)).mp. | 105911 |
| 8 | (chronic$ adj3 bronchiti$).mp | 11116 |
| 9 | exp Adrenal Cortex Hormones/ | 377731 |
| 10 | (adren$ adj1 cort$ adj1 hormone$).mp | 61679 |
| 11 | ICS.mp | 7521 |
| 12 | (inhaled adj1 corticosteroid$).mp | 9027 |
| 13 | (inhaled adj1 steroid$).mp | 2276 |
| 14 | glucocorticoid$.mp. | 102906 |
| 15 | exp FLUTICASONE/ | 2934 |
| 16 | fluticasone.mp. | 4228 |
| 17 | exp BUDESONIDE/ | 4162 |
| 18 | budesonide.mp. | 5720 |
| 19 | exp BECLOMETHASONE | 2940 |
| 20 | beclomethasone.mp. | 3730 |
| 21 | beclometasone.mp. | 215 |
| 22 | exp Mometasone Furoate/ | 684 |
| 23 | mometasone.mp. | 991 |
| 24 | ciclesonide.mp. | 351 |
| 25 | exp TRIAMCINOLONE/ | 8980 |
| 26 | triamcinolone.mp. | 11027 |
| 27 | flunisolide.mp. | 369 |
| 28 | exp Eosinophils/ | 22375 |
| 29 | eosinophil$.mp. | 78401 |
| 30 | 1 or 2 or 3 or 4 or 5 or 6 or 7 or 8 | 262539 |
| 31 | 9 or 10 or 11 or 12 or 13 or 14 or 15 or 16 or 17 or 18 or 19 or 20 or 21 or 22 or 23 or 24 or 25 or 26 or 27 | 416101 |
| 32 | 28 or 29 | 78375 |
| 33 | 30 and 31 and 32 | 2604 |

**Table S5 Embase search strategy (Ovid Embase 1974 to 2019 Week 28)**

| 1 | exp chronic obstructive lung disease/ | 112298 |
| --- | --- | --- |
| 2 | emphysema$.mp. | 46729 |
| 3 | copd.mp. | 74747 |
| 4 | coad.mp. | 376 |
| 5 | cobd.mp. | 16 |
| 6 | (obstruct$ adj3 (pulmonary or lung$ or airway$ or airflow$ or bronch$ or respirat$)).mp. | 181152 |
| 7 | (chronic$ adj3 bronchiti$).mp | 19213 |
| 8 | exp corticosteroid/ | 892507 |
| 9 | (adren$ adj1 cort$ adj1 hormone$).mp | 3253 |
| 10 | ICS.mp | 14447 |
| 11 | (inhaled adj1 corticosteroid$).mp | 14644 |
| 12 | (inhaled adj1 steroid$).mp | 3529 |
| 13 | exp glucocorticoid/ | 683524 |
| 14 | glucocorticoid$.mp. | 126474 |
| 15 | exp fluticasone/ | 7555 |
| 16 | fluticasone.mp. | 16991 |
| 17 | exp budesonide/ | 19075 |
| 18 | budesonide.mp. | 20958 |
| 19 | exp beclomethasone/ | 7193 |
| 20 | beclomethasone.mp. | 3898 |
| 21 | beclometasone.mp. | 14190 |
| 22 | exp mometasone furoate/ | 4459 |
| 23 | mometasone.mp. | 4636 |
| 24 | exp ciclesonide/ | 1428 |
| 25 | ciclesonide.mp. | 1476 |
| 26 | exp triamcinolone/ | 13794 |
| 27 | triamcinolone.mp. | 28561 |
| 28 | exp flunisolide/ | 2378 |
| 29 | flunisolide.mp. | 2417 |
| 30 | exp eosinophil/ | 43093 |
| 31 | eosinophil$.mp. | 118166 |
| 32 | 1 or 2 or 3 or 4 or 5 or 6 or 7 | 239916 |
| 33 | 8 or 9 or 10 or 11 or 12 or 13 or 14 or 15 or 16 or 17 or 18 or 19 or 20 or 21 or 22 or 23 or 24 or 25 or 26 or 27 or 28 or 29 | 923553 |
| 34 | 30 or 31 | 118166 |
| 35 | 32 and 33 and 34 | 2053 |

**Table S6 Web of science (1900 – 2019)**

| 1 | TS=("chronic obstructive pulmonary disease") | 39572 |
| --- | --- | --- |
| 2 | TS=(copd) | 49515 |
| 3 | TS=(coad) | 514 |
| 4 | TS=(chronic obstructive pulmonary disease) | 46282 |
| 5 | TS=(bronchitis) | 18890 |
| 6 | TS=(emphysema) | 21533 |
| 7 | TS=(adrenal cortex hormone*) | 3199 |
| 8 | TS=(glucocorticoid*) | 79053 |
| 9 | TS=(corticosteroid*) | 91793 |
| 10 | TS=(inhaled corticosteroid*) | 12549 |
| 11 | TS=(inhaled steroid*) | 4405 |
| 12 | TS=(ICS) | 20632 |
| 13 | TS=(fluticasone) | 7435 |
| 14 | TS=(budesonide) | 8438 |
| 15 | TS=(beclomethasone) | 4277 |
| 16 | TS=(beclometasone) | 251 |
| 17 | TS=(mometasone) | 1288 |
| 18 | TS=(ciclesonide) | 444 |
| 19 | TS=(triamcinolone) | 8462 |
| 20 | TS=(flunisolide) | 358 |
| 21 | TS=(eosinophil*) | 71291 |
| 22 | #6 OR #5 OR #4 OR #3 OR #2 OR #1 | 98483 |
| 23 | #20 OR #19 OR #18 OR #17 OR #16 OR #15 OR #14 OR #13 OR #12 OR #11 OR #10 OR #9 OR #8 OR #7 | 198176 |
| 24 | #23 AND #22 AND #21 | 761 |

**Table S7 Cochrane CENTRAL (inception to 10.7.19)**

| 1 | ("chronic obstructive pulmonary disease"):ti,ab,kw | 7771 |
| --- | --- | --- |
| 2 | ("COPD"):ti,ab,kw | 12511 |
| 3 | ("COAD"):ti,ab,kw | 60 |
| 4 | ("emphysema"):ti,ab,kw | 1191 |
| 5 | (cobd):ti,ab,kw | 1 |
| 6 | ("lung diseases, obstructive"):ti,ab,kw | 2553 |
| 7 | ("chronic bronchitis"):ti,ab,kw | 1503 |
| 8 | #1 OR #2 OR #3 OR #4 OR #5 OR #6 OR #7 | 17125 |
| 9 | ("inhaled corticosteroid*"):ti,ab,kw | 1678 |
| 10 | ("inhaled steroid*"):ti,ab,kw | 352 |
| 11 | ("glucocorticoid*"):ti,ab,kw | 3002 |
| 12 | (ICS):ti,ab,kw | 1949 |
| 13 | ("adrenal cortex hormone"):ti,ab,kw | 0 |
| 14 | ("fluticasone"):ti,ab,kw | 4474 |
| 15 | ("budesonide"):ti,ab,kw | 3936 |
| 16 | ("beclomethasone"):ti,ab,kw | 2087 |
| 17 | ("beclometasone"):ti,ab,kw | 628 |
| 18 | ("mometasone"):ti,ab,kw | 974 |
| 19 | ("ciclesonide"):ti,ab,kw | 449 |
| 20 | ("triamcinolone"):ti,ab,kw | 2359 |
| 21 | ("flunisolide"):ti,ab,kw | 229 |
| 22 | #9 OR #10 OR #11 OR #12 OR #13 OR #14 OR #15 OR #16 OR #17 OR #18 OR #19 OR #20 OR #21 | 17022 |
| 23 | ("eosinophil*"):ti,ab,kw | 2164 |
| 24 | #8 AND #22 AND #23 | 64 |

**Table S8 CINAHL search (Inception to 10.7.19)**

| 1 | "copd" | 8206 |
| --- | --- | --- |
| 2 | "chronic obstructive pulmonary disease" | 7412 |
| 3 | "chronic bronchitis" | 602 |
| 4 | (MH "Pulmonary Disease, Chronic Obstructive") | 10215 |
| 5 | (MH "Emphysema") | 1288 |
| 6 | "emphysema" | 2229 |
| 7 | (MH "Lung Diseases, Obstructive") | 3245 |
| 8 | 1 OR 2 OR 3 OR 4 OR 5 OR 6 OR 7 | 18105 |
| 9 | (MH "Adrenal Cortex Hormones") | 8114 |
| 10 | ""adrenal cortex hormones"" | 8120 |
| 11 | "glucocorticoid*" | 5082 |
| 12 | ""inhaled corticosteroid*"" | 1815 |
| 13 | "ICS" | 812 |
| 14 | ""inhaled steroid*"" | 394 |
| 15 | (MH "Fluticasone") | 286 |
| 16 | "fluticasone" | 749 |
| 17 | (MH "Budesonide") | 570 |
| 18 | "budesonide" | 793 |
| 19 | (MH "Beclomethasone") | 258 |
| 20 | "beclomethasone" | 373 |
| 21 | "beclometasone" | 22 |
| 22 | "mometasone" | 139 |
| 23 | "ciclesonide" | 66 |
| 24 | (MH "Triamcinolone") | 610 |
| 25 | "triamcinolone" | 798 |
| 26 | "flunisolide" | 35 |
| 27 | 9 OR 10 OR 11 OR 12 OR 13 OR 14 OR 15 OR 16 OR 17 OR 18 OR 19 OR 20 OR 21 OR 22 OR 23 OR 24 OR 25 OR 26 | 15806 |
| 28 | (MH "Eosinophils") | 841 |
| 29 | "eosinophil*" | 3437 |
| 30 | 28 OR 29 | 3437 |
| 31 | 8 AND 27 AND 30 | 40 |
